# An oral health module for use in noncommunicable disease risk factor surveys

**DOI:** 10.2471/BLT.25.295572

**Published:** 2026-08-20

**Authors:** Nicole Rendell, Habib Benzian, Richard G Watt, Leanne Riley, Eugenio Beltrán, Eduardo Bernabe, Jonathan Broadbent, Moréniké O Foláyan, Heba Fouad, Nicolas Giraudeau, Nalika Gunawardena, Yuriko Harada, Anja Heilmann, Carolina Hommes, Yuka Makino, Ricardo Martinez, Sudeshni Naidoo, Marco A Peres, Waranuch Pitiphat, Maha El Tantawi, Georgios Tsakos, Elena Tsoy, Renato Venturelli, Benoit Varenne

**Affiliations:** a Noncommunicable Diseases and Mental Health Department, World Health Organization, 20 Avenue Appia, 1211 Geneva 27, Switzerland.; b WHO Collaborating Centre for Oral Health, University of the Western Cape, Cape Town, South Africa.; c WHO Collaborating Centre for Oral Health Inequalities and Public Health, University College London, London, England.; d WHO Collaborating Centre for Quality Improvement and Evidence-based Dentistry, New York University, New York, United States of America (USA).; e Centre for Dental Public Health and Primary Care, Queen Mary University of London, London, England.; f Department of Oral Sciences, University of Otago, Dunedin, New Zealand.; g Department of Child Dental Health, Obafemi Awolowo University, Ile-Ife, Nigeria.; h Department of Noncommunicable Diseases and Mental Health, WHO Regional Office for the Eastern Mediterranean, Cairo, Egypt.; i Centre d’Etude Politique et SociaLe (CEPEL), Centre National de Recherche Scientifique (CNRS), University of Montpellier, Montpellier, France.; j Department of Health Promotion, Disease Prevention & Control, WHO Regional Office for South-East Asia, New Delhi, India.; k Healthy Life Course Integrated Approach, WHO Regional Office for the Americas, Washington, DC, USA.; l Noncommunicable Diseases, WHO Regional Office for Africa, Brazzaville, Republic of Congo.; m Health Services Research and Population Health Programme, Duke-NUS Medical School, Singapore, Singapore.; n Faculty of Dentistry, Khon Kaen University, Khon Kaen, Thailand.; o Department of Pediatric Dentistry and Dental Public Health, Alexandria University, Alexandria, Egypt.; p Noncommunicable Diseases Management, WHO Regional Office for Europe, Copenhagen, Denmark.

## Abstract

The World Health Organization (WHO) *Global strategy and action plan on oral health 2023–2030* emphasizes the need to strengthen oral health information systems, including surveillance, to support public health action. Objective 5 of the *Global strategy and action plan on oral health 2023–2030* calls for reliable, timely and policy-relevant data to inform decision-making, promote accountability and monitor progress, in other words, surveillance of oral diseases. However, most countries face challenges in identifying and applying practical tools to generate such population-level data. Until recently, the WHO STEPwise approach to noncommunicable disease risk factor surveillance (STEPS) included an oral health module that relied on self-reported data on perceived oral health and oral health-seeking behaviour. However, this module is outdated. In response to growing global demand and policy needs, the new STEPS oral health module updated the self-reported questionnaire and added a clinical assessment component that can be done by oral or non-oral care workers after a short training. This paper presents the rationale, development process, structure and policy implications of the updated module. The paper provides an outline of the updated module and evidence to support this new approach as a practical, scalable and aligned solution to one of the most persistent gaps in noncommunicable disease surveillance. As countries move towards universal health coverage and integration of oral health into broader noncommunicable disease strategies, the updated WHO STEPS oral health module is a timely and necessary improvement in surveillance of noncommunicable diseases and their risk factors.

## Résumé

Le Plan d’action mondial pour la santé bucco-dentaire 2023–2030 de l’Organisation mondiale de la Santé (OMS) souligne la nécessité de renforcer les systèmes d’information sur la santé bucco-dentaire, y compris la surveillance, afin de soutenir l’action en matière de santé publique. L’objectif nº 5 du Plan d’action mondial pour la santé bucco-dentaire 2023–2030 préconise la collecte de données fiables, actualisées et pertinentes pour l’élaboration de politiques afin d’éclairer la prise de décision, de favoriser la responsabilisation et de faire le suivi des progrès, autrement dit d’assurer la surveillance des maladies bucco-dentaires. Toutefois, la plupart des pays rencontrent des difficultés pour identifier et mettre en œuvre des outils pratiques permettant de générer de telles données à l’échelle d’une population. Jusqu’à récemment, l’approche STEPwise de l’OMS pour la surveillance des facteurs de risque des maladies non transmissibles (STEPS) comprenait un module consacré à la santé bucco-dentaire qui s’appuyait sur des données autodéclarées concernant la perception de la santé bucco-dentaire et les comportements en matière de recours aux soins bucco-dentaires. Ce module est désormais obsolète. En réaction à une demande mondiale croissante et aux besoins en matière de politiques publiques, le nouveau module de santé bucco-dentaire du programme STEPS de l’OMS a mis à jour le questionnaire d’auto-évaluation et intègre désormais un volet d’évaluation clinique à destination des professionnels de la santé bucco-dentaire ou d’autres disciplines, moyennant une brève formation. Cet article présente la justification, le processus d’élaboration, la structure et les implications politiques du module mis à jour. Il esquisse le module actualisé ainsi que des données étayant cette nouvelle approche, présentée comme une solution pratique, évolutive et cohérente pour combler l’une des lacunes les plus persistantes en matière de surveillance des maladies non transmissibles. Alors que les pays s’orientent vers une couverture sanitaire universelle et l’intégration de la santé bucco-dentaire dans des stratégies plus larges de lutte contre les maladies non transmissibles, le module actualisé du programme STEPS de l’OMS consacré à la santé bucco-dentaire constitue une amélioration opportune et nécessaire de la surveillance des maladies non transmissibles et de leurs facteurs de risque.

## Resumen

El Plan de Acción Mundial sobre Salud Bucodental 2023–2030 de la Organización Mundial de la Salud (OMS) destaca la necesidad de reforzar los sistemas de información sobre salud bucodental, incluida la vigilancia, para apoyar las medidas de salud pública. El Objetivo 5 del Plan de Acción Mundial sobre Salud Bucodental 2023–2030 exige datos fiables, oportunos y pertinentes para las políticas con el fin de fundamentar la toma de decisiones, promover la rendición de cuentas y controlar el progreso; en otras palabras, la vigilancia de las enfermedades bucodentales. Sin embargo, la mayoría de los países afrontan desafíos para identificar y aplicar las herramientas prácticas que permitan generar este tipo de datos a nivel poblacional. Hasta hace poco tiempo, el enfoque STEPwise de la OMS relativo a la vigilancia de los factores de riesgo de las enfermedades no transmisibles (STEPS) incluía un módulo de salud bucodental que se basaba en datos declarados por los propios participantes sobre la percepción de su salud bucodental y sus conductas de búsqueda de atención odontológica. Sin embargo, este módulo se ha quedado obsoleto. En respuesta al crecimiento de la demanda global y de las necesidades políticas, el nuevo módulo de salud bucodental STEPS de la OMS actualizó el cuestionario de autoinforme e incluye un componente de evaluación clínica que puede ser realizado por el personal sanitario, sea o no de salud bucodental, tras una breve capacitación. Este artículo presenta la justificación, el proceso de desarrollo, la estructura y las implicaciones políticas del módulo actualizado. El artículo proporciona un resumen del módulo actualizado y la evidencia que respalda este nuevo enfoque como una solución práctica, escalable y orientada para resolver una de las brechas más persistentes en lo relativo a la vigilancia de las enfermedades no transmisibles. A medida que los países avanzan hacia la cobertura sanitaria universal y la integración de la salud bucodental en estrategias más amplias contra las enfermedades no transmisibles, el módulo actualizado de salud bucodental STEPS de la OMS constituye una mejora oportuna y necesaria para la vigilancia de las enfermedades no transmisibles y sus factores de riesgo.

## ملخص

تركز خطة العمل العالمية لصحة الفم (2023-2030)، الصادرة عن منظمة الصحة العالمية (WHO) على الحاجة لتعزيز نظم المعلومات المتعلقة بصحة الفم، بما في ذلك أنظمة الرصد، بهدف دعم إجراءات الصحة العامة. ويدعو الهدف الخامس من خطة العمل العالمية لصحة الفم (2023-2030)، إلى توفير بيانات موثوقة وفي الوقت المناسب وذات صلة بالسياسات، وذلك لتوجيه عملية صنع القرار وتعزيز المساءلة ورصد التقدم المحرز؛ وبعبارة أخرى، إجراء رصد لأمراض الفم. ومع ذلك، تواجه معظم الدول تحديات في تحديد وتطبيق أدوات عملية لتجميع مثل هذه البيانات على مستوى السكان. وحتى وقت قريب، كان أسلوب منظمة الصحة العالمية لرصد عوامل خطر الأمراض غير المعدية (STEPS) تتضمن وحدة خاصة بصحة الفم تعتمد على بيانات الإفصاح الذاتي حول الحالة المتصورة لصحة الفم والسلوكيات المتعلقة بطلب الرعاية الصحية للفم. إلا أن هذه الوحدة أصبحت عتيقة. واستجابةً للطلب العالمي المتزايد والاحتياجات المتعلقة بالسياسات، قامت وحدة صحة الفم (STEPS) التابعة لمنظمة الصحة العالمية، بتحديث الاستبيان الذي يعتمد على الإفصاح الذاتي، كما اشتملت على مكونات التقييم الإكلينيكي التي يمكن تنفيذها بعد تلقي تدريب قصير، بواسطة العاملين أو غير العالمين في مجال صحة الفم. وتستعرض هذه الورقة المبدأ، وعملية التطوير، والهيكل، والآثار المترتبة على السياسات فيما يخص هذه الوحدة التي تم تحديثها. كما تقدم الورقة لمحة عامة عن الوحدة التي تم تحديثها والأدلة الداعمة لهذا الأسلوب الجديد باعتباره حلاً عملياً وقابلاً للتوسع ومتوافقاً مع الجهود الرامية لسد واحدة من أكثر الفجوات استمراراً في مجال رصد الأمراض غير المعدية. وفي ظل سعي الدول نحو تحقيق التغطية الصحية الشاملة ودمج خدمات صحة الفم ضمن الاستراتيجيات الأوسع نطاقاً لمكافحة الأمراض غير المعدية، فإن وحدة صحة الفم التي تم تحديثها ضمن نظام STEPS التابع لمنظمة الصحة العالمية، تُعد تحسيناً ضرورياً وفي وقته المناسب لأنظمة رصد الأمراض غير المعدية وعوامل الخطر المرتبطة بها.

## 摘要

世卫组织 (WHO)《口腔卫生全球行动计划（2023-2030 年）》强调，需加强口腔卫生信息系统（包括监测系统）建设，以支持开展公共卫生行动。《口腔卫生全球行动计划（2023-2030 年）》目标 5 要求，提供可靠、及时的政策相关数据，以支持决策制定、强化问责制度并监测进展，即口腔疾病监测。然而，大多数国家在识别并应用实用工具来生成此类人群层面数据方面仍面临着诸多挑战。直到最近，世卫组织慢性非传染性疾病危险因素监测阶梯式方法 (STEPS) 仍在采用一种口腔卫生模块，以利用自我报告数据来获取感知口腔卫生状况和口腔卫生求医行为相关信息。但是，该模块现已过时。为了应对不断升级的全球需求和不断发展的政策需要，新推出的世卫组织 STEPS 口腔卫生模块更新了自我报告问卷，并纳入了一个临床评估环节，口腔或非口腔卫生工作者经短期培训后即可完成相应操作。本文介绍了更新版模块的理论依据、发展过程、结构和政策启示。本文概述了更新版模块，并提供了相应证据，以证明这种作为切实可行、可扩展且协调一致的解决方案的新方法，可用于填补非传染性疾病监测领域长期存在的最大缺口之一。随着各国致力于实现全民健康覆盖并将口腔卫生纳入范围更广的非传染性疾病战略，更新版世卫组织 STEPS 口腔卫生模块是对非传染性疾病及相关危险因素监测系统的及时、必要改进。

## Резюме

В Глобальном плане действий Всемирной организации здравоохранения (ВОЗ) по охране здоровья полости рта на 2023–2030 годы подчеркивается необходимость укрепления информационных систем в этой области, включая эпидемиологический надзор, в целях поддержки мер общественного здравоохранения. Задача 5 Глобального плана действий по охране здоровья полости рта на 2023–2030 годы требует наличия надежных, своевременных и актуальных для стратегии данных для информационного обеспечения процессов принятия решений, повышения подотчетности и мониторинга прогресса, иными словами для эпидемиологического надзора за заболеваниями полости рта. Однако большинство стран сталкиваются с трудностями при определении и применении практических инструментов для получения таких данных на уровне населения. До недавнего времени поэтапный подход ВОЗ к эпидемиологическому надзору за факторами риска неинфекционных заболеваний (STEPS) включал модуль по охране здоровья полости рта, который опирался на данные самоотчетов о воспринимаемом состоянии здоровья полости рта и поведении в отношении обращения за медицинской помощью при проблемах здоровья полости рта. Однако данный модуль устарел. В ответ на растущий глобальный спрос и стратегические потребности в новом модуле ВОЗ STEPS по охране здоровья полости рта был обновлен опросник на основе самоотчетов, а также был включен компонент клинической оценки, которую могут проводить стоматологические или нестоматологические медицинские работники после краткого обучения. В настоящей статье представлены: обоснование, процесс разработки, структура и последствия применения обновленного модуля для стратегии. В статье приводится краткое описание обновленного модуля и доказательства в поддержку этого нового подхода как практичного, масштабируемого и согласованного решения для устранения одного из наиболее стойких упущений в эпидемиологическом надзоре за неинфекционными заболеваниями. По мере того как страны продвигаются к всеобщему охвату услугами здравоохранения и интеграции вопросов охраны здоровья полости рта в более широкие стратегии по борьбе с неинфекционными заболеваниями, обновленный модуль ВОЗ STEPS по охране здоровья полости рта представляет собой своевременное и необходимое усовершенствование в сфере эпидемиологического надзора за неинфекционными заболеваниями и факторами их риска.

## Oral disease surveillance

Oral diseases and conditions (herein collectively referred to as oral diseases) are the most prevalent noncommunicable diseases, affecting an estimated 3.7 billion people worldwide.^1^ Despite being largely preventable, this high burden of oral diseases has continued to rise over the past 30 years, with prevalence indicating widespread social and economic inequalities.^2^ At an individual level, untreated oral disease may cause pain, reduced quality of life, lost school days, disruption to family life, decreased work productivity and, in some cases, can lead to severe complications and death.^3^ Yet the availability of surveillance data on oral diseases is limited.

In recent years, integration of oral health into the broader noncommunicable disease agenda has gained momentum, with growing recognition that oral diseases are a major public health issue and that comprehensive, people-centred primary health care is the pathway to achieving universal health coverage (UHC). Within primary care, task-shifting and interprofessional teams are being used to expand access to oral care, for example by training nurses or community health workers to deliver essential oral health interventions, such as topical fluoride application. This development underscores the need to embed essential oral health services into prevention, care, surveillance and financing mechanisms across the life course to advance equity and population health.

The 2021 World Health Assembly Resolution on oral health (WHA74.5) mandated the development and adoption by World Health Organization (WHO) Member States of the first *Global strategy and action plan on oral health 2023–2030*.^4^ Together, these strategic documents define a renewed global oral health agenda built on six strategic objectives to advance affordable and accessible oral health care within the vision of UHC (Box 1). Objective 5 of the *Global strategy and action plan on oral health 2023–2030*^4^ prioritizes effective surveillance systems to monitor oral diseases, service access and equity, inform policy, optimize resource allocation and track progress and outcomes over time. The actions focus on integrating oral health data with national-level noncommunicable disease surveillance and embracing digital technologies to support timely and accurate data collection.

Box 1 **Strategic objectives (and purpose) of the *Global strategy and action plan on oral health 2023–2030***

1. Oral health governance (to improve political and resource commitment to oral health, strengthen leadership and create win–win partnerships within and outside the health sector).
2. Oral health promotion and oral disease prevention (to enable all people to achieve the best possible oral health and address the social and commercial determinants and risk factors of oral diseases and conditions).
3. Health workforce (to develop innovative workforce models and revise and expand competency-based education to respond to population oral health needs).
4. Oral health care (to integrate essential oral health care and ensure related financial protection and essential supplies in primary health care).
5. Oral health information systems (to enhance surveillance and health information systems to provide timely and relevant feedback on oral health to decision-makers for evidence-based policy-making).
6. Oral health research agendas (to create and continuously update context- and needs-specific research focused on the public health aspects of oral health).

Source: World Health Organization; 2024.^4^

Surveillance of oral diseases at both facility-based and population-based levels, is underdeveloped in most countries. Typically, population data on oral diseases are gathered through national oral health surveys which are costly, conducted infrequently, absent in some countries, or insufficiently representative of the entire population. Previous WHO guidance aimed to standardize the approach to national oral health surveys through the *Oral health surveys: basic methods, 5th edition*, but the suitability of such surveys for policy development and outcome evaluation is limited.^5^ These surveys typically focus on narrow age groups and rely on technical epidemiological measures, without a rigorous sampling method to ensure population representativeness. For most countries, the surveys are unaffordable and isolated from other public health surveillance programmes; they serve mainly research purposes rather than integrated surveillance and monitoring.

Much of the data currently used to estimate the global burden of oral diseases, particularly from the Global Burden of Disease (GBD) study, relies on the identification of high-quality primary research studies on oral diseases burden and advanced data modelling.^6^ Therefore, as GBD estimates will inevitably depend on primary sources, developing more efficient approaches to conducting primary studies based on representative population samples would help strengthen the data quality of comparable country data on oral diseases globally.^7^ However, GBD data alone may be insufficient for national planning and lack the local specificity needed to guide implementation strategies or assess policy impact.

Recognizing the need for improvement, WHO initiated a comprehensive revision of the oral health module of the WHO STEPwise approach to noncommunicable disease risk factor surveillance (STEPS). The new version contains a shorter and more focused questionnaire and includes a brief clinical assessment component that trained oral or non-oral care workers can conduct. This paper introduces the updated module, discusses its development process, details its structure and explores its potential contribution to national and global monitoring of oral diseases as part of noncommunicable disease surveillance.

## Rationale for the update

WHO STEPS is a standardized method for collecting data on behavioural and biological risk factors of noncommunicable diseases. STEPS adopts probabilistic sampling to ensure national representativeness and weighted data. This sampling is achieved by a multistage cluster design, with the households surveyed being randomly selected from within each cluster. The STEPS survey is designed for use with people aged 18−69 years and gathers essential information through questionnaires, physical measurements and biochemical assessments. The core module is supported by optional modules that countries can include based on their specific public health priorities and capacity. These optional modules may also include questionnaires and physical measurements. The approach enables comparability across populations and time, while allowing customization to meet national needs.^8^ Even when collected at multiyear intervals, STEPS surveys provide essential anchor points for trend analysis, benchmarking and evaluation of policy impact.

From 2000 to 2023, the previous STEPS optional module on oral health was included by 46 of the 136 countries that completed the STEPS survey. However, this earlier version had limitations. The module relied solely on self-reported data on perceived oral health and oral health-care-seeking behaviour, provided limited policy-relevant information and lacked alignment with global indicators. These limitations made the module less useful for countries and restricted its analytical use.

WHO recognized that updating the oral health module within the STEPS framework offered the most practical opportunity to improve the collection of population-level data on oral diseases. Therefore, the process to revise the STEPS oral health module was undertaken with three strategic goals for direct policy relevance: (i) to focus the questionnaire on core oral health and access to care indicators; (ii) to add a clinical component that would be feasible in household survey settings; and (iii) to align with both the monitoring framework of the *Global strategy and action plan on oral health 2023–2030* and the requirements of the GBD study. This approach reflects a shift from research-oriented oral health surveys towards policy-oriented surveillance tools designed for routine integration within national health information systems.

## Development of the updated module

The development of the updated module followed a rigorous, consultative process. The WHO Oral Health Programme and the Surveillance, Monitoring and Reporting Unit, both within the WHO Noncommunicable Diseases Department, jointly undertook a preliminary review of the existing module.

In November 2023, WHO convened an expert workshop in London, United Kingdom of Great Britain and Northern Ireland, with in-person and online attendance and hosted by the WHO Collaborating Centre for Oral Health Inequalities and Public Health at University College London. The workshop brought together oral epidemiologists, surveillance experts, GBD collaborators, and staff from WHO headquarters and regional offices. The expert panel reviewed draft proposals for both the updated questionnaire and the new clinical component. Participants discussed the selection of indicators, methodological considerations and practical aspects of implementation.^9^

Consensus was reached on several key elements. First, the questionnaire would be reduced from 14 questions to five questions: self-rated oral health; the functional and psychosocial impacts of oral disease; recent patterns of care-seeking behaviour; barriers to access; and use of fluoride toothpaste. Second, the question on the use of fluoride toothpaste would be strengthened through a showcard-based verification process where respondents would be presented with a selection of locally available toothpaste brands to help identify which one they use. Fluoride toothpaste is widely available and its use is well-suited to data collection through population-based surveys. By contrast, water fluoridation (another way to deliver fluoride) is typically monitored through administrative and regulatory data systems rather than population-based surveys. Accordingly, data on fluoridation coverage and concentration are obtained directly from national authorities and interpreted alongside STEPS findings. Third, the clinical assessment would focus on four indicators: tooth count; untreated caries (using the stop-after-first-encounter approach);^10^^–^^12^ caries-related complications (assessed using a simplified pulp involvement, ulceration, fistula or abscess index);^13^^,^^14^ and identification of any other soft tissue lesion of the lip or oral cavity. All clinical assessments would rely on visual inspection, allowing data collectors who are not oral care workers to conduct these assessments safely and effectively in field settings, as part of the standard STEPS training procedures.

During the workshop discussion, application of digital technologies using a portable intraoral camera to record videos or photographs was explored. However, the group could not identify a scalable tool and concluded that further research was needed on the reliability and affordability of emerging tools in diagnosing oral diseases for surveillance purposes.^9^ The outcomes of the workshop are reported elsewhere.^9^

## Updated module

The updated STEPS oral health module consists of two components: a structured questionnaire and a clinical assessment (Table 1 and Table 2).^8^

**Table 1:** WHO STEPwise approach to noncommunicable disease risk factor surveillance, oral health module questionnaire

Question	Theme	Purpose and rationale
O1. How do you rate your oral health (conditions of teeth, gums and mouth) right now?	Self-rated oral health	• Provides a validated global item correlated with objective oral health status and quality of life^15^^–^^17^ • Is simple, widely comparable and in alignment with self-rated health measures in other sectors
O2. During the past twelve months, did you experience one or more of the following issues because of your teeth, gums or mouth? *Please mark all that apply: pain; difficulty eating food; difficulty speaking; avoided smiling; difficulty doing other usual activities; none; other; don't know*	Functional and psychosocial impacts	• Reflects oral health-related quality of life, i.e. the impact of oral conditions on daily life of people^18^^,^^19^ • Has direct relevance to economic productivity, social well-being and daily functioning
O3. During the past twelve months, have you used health-care services because of a problem with your teeth, gums or mouth?	Use of health-care services	• Is aligned with universal health coverage indicators and access to essential care^20^^–^^22^ • Indicates structural and system access to health-care services • Enables monitoring of recent service use
O4. What were the reasons for not using health-care services? *Please mark all that apply: no service available; too expensive; service too far away; poor service quality; fear (afraid of dental care); no appointment available; relief from self-medication, other*	Barriers to care	• Explores access barriers, inequity and systemic challenges^20^^–^^22^ • Helps identify actionable problems in service delivery and use
O5. Please indicate which of the toothpastes shown do you use to clean your teeth? (showcard method to verify fluoride content)	Fluoride use	• Links self-report with visual verification of fluoride toothpaste use^8^^,^^23^^,^^24^ • Is practical for field use

WHO: World Health Organization.

Note: see reference for full details of the questions.

Source: WHO, 2026.^8^

**Table 2:** WHO STEPwise approach to noncommunicable disease risk factor surveillance, oral health module clinical assessment

Clinical component	Description	Rationale and alignment
O6. Tooth count	Examiner visually counts and categorizes natural teeth: 0, 1–9, 10–19 or ≥ 20	• Is aligned with WHO and GBD metrics on tooth loss and edentulism (*Global strategy and action plan on oral health 2023–2030 *complementary indicator B7)^4^ • Functional dentition is a core component of oral health • Is simple, meaningful and related to quality of life and nutrition
O7. Presence of untreated dental caries	Using Stop-After-First-Encounter method, examiner checks for visible cavitated lesions and stops when one lesion is identified	• Provides prevalence estimate for untreated caries (*Global strategy and action plan on oral health 2023–2030* complementary indicators B4/B5)^4^ • Reduces examination time • Is GBD-aligned and feasible for field use without probing or complex diagnosis
O8. Presence of severe dental caries	If caries is found, examiner assesses whether it has progressed to pulp involvement, ulceration, fistula or abscess. Stops after first sign	• Identifies advanced cases of decay and dental infection • Uses pulp involvement, ulceration, fistula or abscess which is recognized in WHO global oral health monitoring as a key indicator for disease severity (*Global strategy and action plan on oral health 2023–2030* complementary indicator B3)^4^
O9. Presence of severe pain, swelling, damage or unusual lesion in or around the mouth	Examiner assesses the presence of severe pain, swelling, damage or unusual lesion in or around the mouth based on a visual inspection of the soft tissues in and around the mouth, and the declaration of the participant	• Identifies any other soft tissue lesions that may require referral for further investigation

GBD: Global Burden of Disease; WHO: World Health Organization.

Source: WHO, 2026.^8^

The questionnaire includes five questions that assess the respondent’s perceived oral health,^15^^–^^17^ experiences of the impact of oral health issues on daily functioning (such as pain and difficulty eating),^18^^,^^19^ and the use of health-care services when experiencing an oral health problem including the reason(s) for not accessing care.^20^^–^^22^ The final question asks about toothpaste and presents showcards of locally available toothpaste brands. The system has coded which of these toothpastes contain fluoride and which ones do not to enable analysis on the use of fluoride toothpaste, which is known to be effective at a concentration of 1000–1500 parts per million (ppm).^8^^,^^23^^,^^24^ The classification of fluoride toothpaste is based on information provided on the packaging, supported by the knowledge of national oral health leaders within the STEPS implementation team. This approach reflects real-world regulatory environments, where packaging serves as the primary source of consumer information, and prioritizes feasibility and external validity within population-based surveys. This approach is a means of monitoring access to and use of fluoridated toothpaste in a scalable way across countries, although some non-differential exposure misclassification may occur.

The clinical component has a structured sequence. First, the examiner counts the number of teeth present and records whether the participant has zero, 1–9, 10–19, or 20 or more teeth. Next, the examiner systematically checks each tooth for the presence of cavitated carious lesions using the stop-after-first-encounter approach.^10^^–^^12^ The examination is stopped after the first carious lesion is detected and recorded. If caries is detected, restarting the cycle from the beginning, the examiner then assesses each tooth for any signs of pulp involvement, ulceration, fistula or abscess visible in the mouth, again using the stop-after-first-encounter approach: if the examiner detects a sign of pulp involvement, ulceration, fistula or abscess, the examination is stopped and the presence of severe caries is recorded.^10^^–^^14^ Finally, the examiner assesses the presence of severe pain, swelling, damage or unusual lesions in or around the mouth based on the participant's declaration and a visual inspection of the soft tissues in and around the mouth, and records the finding.

## Implementation of the module

Following consensus on the content and structure of the updated STEPS oral health module, the module then underwent an internal WHO review and field testing process. The results of the field tests were used to refine both the questionnaire and clinical assessment protocols before finalization. Implementation of the questionnaire section remained the same as with the previous STEPS oral health module. The clinical component however is a new addition designed to be implemented by trained oral and non-oral care workers in household settings. WHO provides training on the oral health module as part of the STEPS comprehensive training package for field data collectors. The training emphasizes visual recognition of clinical signs, appropriate use of light sources and respectful interaction with participants, including obtaining informed consent and providing referral information as needed. As the oral health module is implemented as part of a broader STEPS survey, sampling, data quality checks and other logistical aspects are managed by the in-country team responsible for planning the survey with the support of the WHO STEPS team.^8^

Since the updated oral health module was first released for field testing in 2024, it has been implemented as part of STEPS surveys in eight countries. Current engagement and feedback from WHO Member States indicates growing interest in adopting the updated oral health module.

## Advances and innovations

The updated STEPS oral health module represents a methodological innovation, shifting from episodic, research-driven surveys towards periodic, integrated surveillance within broader noncommunicable disease monitoring systems. The new oral health module offers an updated self-reported questionnaire and a practical clinical assessment that can be conducted by oral and non-oral care workers with training. The module represents an important step forward in expanding surveillance coverage, especially in resource-constrained settings, while substantially reducing the cost and logistical complexity associated with isolated and full-scale national oral health surveys.

Furthermore, the module’s compatibility with the broader STEPS survey structure provides opportunities for integrated data analysis. By connecting oral health data with other STEPS modules, such as diabetes, hypertension, nutrition and tobacco use, countries can better understand common risk factors and explore the interrelationships between oral health and general health. This data-driven approach enhances the rationale for coordinated, cross-sectoral policy development, planning and surveillance to improve population health.

Another important innovation is the module’s alignment with indicators used in the GBD study. By adopting compatible definitions and streamlined assessment protocols, such as the stop-after-first-encounter method for untreated caries and binary pulp involvement, ulceration, fistula or abscess scoring,^10^^–^^14^ the data generated through this module can directly contribute to the GBD estimation process on dental caries. The alignment is further enhanced by the adoption of probabilistic sampling that allows nationally representative estimates. Enabling countries to generate primary data that can be integrated into global disease burden estimates enhances both the visibility and policy utility of national survey efforts in shaping international oral health metrics.

## Beyond the STEPS framework

The STEPS method with its adult-focused surveillance of 18–69-year-olds provides substantial policy value. Adults carry the cumulative burden of oral diseases, including untreated caries and tooth loss, making this age group central to estimating population needs. In addition, adult data are directly relevant for health-system planning, including service delivery, financing and integration within UHC frameworks. The use of a standardized STEPS platform further enables comparability across countries and over time, which is not currently achievable through most school-based surveys.

However, population-level data on oral diseases for people outside this age range are not assessed. While countries may like to expand the survey to younger or older age groups, the current module does not offer guidance or standardization for these applications. Future adaptations of the STEPS tool may revisit the age range in line with evolving needs and experience from early implementation. However, it is important to note that countries are free to adapt core elements of the STEPS oral health module through the inclusion of additional indicators aligned with their national noncommunicable disease surveillance. Similarly, geographically large countries with diverse population groups may benefit from subnational data for policy design and evaluation.

## Limitations

While the updated STEPS oral health module introduces advances in feasibility and policy alignment, there are some limitations. The module is designed to generate consistent and policy-relevant population-level estimates under real-world conditions, rather than to achieve diagnostic precision at the individual level.

The clinical component focuses on dental caries as the most prevalent oral disease globally.^2^^,^^6^ This focus ensures alignment with disease burden estimates and global monitoring frameworks but also reflects a compromise. At present, no validated, simple and reliable method exists for assessing periodontal disease that can be performed without periodontal probing,^9^^,^^25^ although work to develop such a method is ongoing.^26^^–^^28^ Such assessments would require trained oral care workers and specialized instruments, substantially increasing the cost, time required and complexity of implementing the survey. This additional assessment would undermine the module’s goal of use in routine household surveys with the potential for data collection by non-oral care workers.

A second limitation relates to the simplified diagnostic approach adopted for caries and its consequences. The module uses binary scoring through the stop-after-first-encounter method^10^^–^^12^ for caries detection and a simplified pulp involvement, ulceration, fistula or abscess index to identify severe disease.^13^^,^^14^ These approaches do not capture the full range of clinical details typically gathered using indices such as decayed, missing and filled teeth, or the full pulp involvement, ulceration, fistula or abscess scoring system.^13^^,^^14^ The visual-only examination excludes detection of early-stage carious lesions and may also reduce detection sensitivity of subtle soft-tissue or mucosal conditions. This streamlined approach reflects another compromise of large epidemiological surveys where a simplified method is preferred in the interest of feasibility.

These limitations are trade-offs that prioritize feasibility, affordability, reliability and integration. The updated module is a practical alternative to national oral health surveys using the *Oral health surveys: basic methods, 5th edition*^5^ to collect policy-relevant oral health data. Further work is needed to evaluate the module’s performance in various country contexts and settings. Particular emphasis should be given to assessing the diagnostic sensitivity for estimating caries prevalence, evaluating consistency across data collectors with different health-worker profiles and conducting implementation research.

## Conclusion

The updated STEPS oral health module is an important step in the global effort to strengthen surveillance of noncommunicable diseases and associated risk factors. Additionally, the module contributes to improved evidence-informed decision-making towards the *Global strategy and action plan on oral health 2023–2030*’s vision of UHC for oral health. By revising and updating the questionnaire content to make it more policy-relevant and integrating a practical clinical assessment into a standardized household survey framework, the module has the potential to bridge a long-standing gap in surveillance of oral diseases. These changes equip national health information systems with the means to strengthen noncommunicable disease surveillance in a practical, integrated and equity-sensitive way. The updated module provides a practical tool for countries to generate credible and actionable data. The data generated through the module are intended to inform national decision-making processes led by government health agencies, including chief dental officers, noncommunicable disease programme managers and planners responsible for UHC, who are accountable for translating surveillance data and evidence into policy, planning and resource allocation decisions.
